# Image-Segmentation-Guided Fragmentized Steel Scrap Tramp Material Characterization

**DOI:** 10.1007/s40831-026-01569-x

**Published:** 2026-06-24

**Authors:** Yijun Quan, Sanjay Singhal, Zushu Li, Giovanni Montana

**Affiliations:** 1 WMG, University of Warwick, Coventry, CV4 7AL UK; 2 Global Ardour Recycling, Preston, PR2 5BX UK

**Keywords:** Steel scrap recycling, Tramp element analysis, Image segmentation, Mutual learning, Computer vision

## Abstract

**Abstract:**

As the British steel industry transitions to a scrap-based electric arc furnace route for steelmaking, steel scrap quality is becoming increasingly important. The chemical composition of fragmentized scrap, particularly the weight percentage of elements such as copper, strongly governs its suitability for high-quality steelmaking. However, existing chemical characterization techniques are too expensive and operationally impractical to deploy across the entire scrap stream, leaving composition estimates vulnerable to fluctuations in tramp element distributions introduced by unwanted residuals. To enable fast, cost-effective assessment, this work uses copper as an exemplar and investigates image-based classification for tramp element composition estimation for fragmentized scrap. Using commercial-grade scrap, an image dataset was manually curated to capture diverse mixtures of steel and copper fragments, with corresponding weight measurements and segmentation annotations collected for each sample image. A neural network was then trained using image segmentation, with the predicted segmentation maps provided as input to a classifier, achieving $86.67\%$ accuracy in assigning images to the correct copper composition class and demonstrating the feasibility of computer vision methods for tramp element composition analysis in scrap quality monitoring.

**Graphical Abstract:**

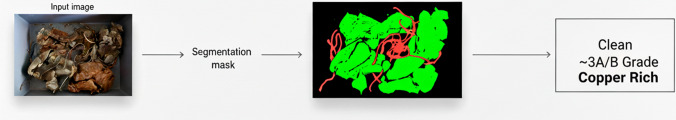

## Introduction

Steel production is a main contributor to the world’s carbon emissions, accounting for about $7\%$ of global greenhouse gas (GHG) emissions and $11\%$ of global carbon dioxide (CO$_{2}$) emissions [1]. The shift from conventional blast furnace (BF)–basic oxygen furnace (BOF) routes to electric arc furnace (EAF) steelmaking represents a key strategy for decarbonizing the steel sector. In the UK, ongoing investments to replace BFs/BOFs with new EAF installations signal a fundamental transformation of the national steel production landscape toward lower-carbon processes. Notably, a scrap-fed EAF has a cradle-to-gate (CTG) CO$_{2}$ emission intensity estimated at $0.27\,{\text {t}}\,{\text {CO}}_{2}/{\text {t}}$ of steel, which is significantly lower than the $1.99\,{\text {t}}\,{\text {CO}}_{2}/{\text {t}}$ from traditional BOF processes [2]. However, this transition further elevates the centrality of scrap in steelmaking, making consistent scrap quality increasingly critical.

Steel scrap quality depends on multiple factors which can directly impact EAF performance. Scrap morphology directly influences melting energy requirements and thus production costs, with smaller pieces melting faster owing to higher surface-area-to-volume ratios. More critically, chemical composition, particularly tramp element concentrations such as copper, tin, and lead, affects both energy efficiency and the mechanical properties of the final steel, where excess levels can compromise ductility and induce brittleness if not controlled. In EAF steelmaking, operators blend different types of steel scrap in controlled ratios to achieve the desired chemical composition. Unanticipated compositional variations in any input stream can yield off-specification steel, increase production costs, reduce yields, and introduce variability in the final mechanical properties. According to the British Metals Recycling Association (BMRA), steel scraps in the UK are classified into 36 main grades [3]. While some grades, such as grade 12B (new production profiles) and grade 8A (new loose light steel cuttings), exhibit relatively consistent composition such that statistically representative readings can be obtained from a small quantity without testing the entire batch, other forms are subject to much larger compositional variation. Among these, grades 3A and 3B (fragmentized old light steel, differing in dimensions) are among the most abundant, with grade 3B alone contributing approximately $28.5\%$ of steel scrap produced in the UK [4]. Therefore, expanding the use of 3A/3B fragmentized scrap would significantly improve the overall recyclability of steel scrap.

Although guidelines specify tramp element limits for fragmentized steel scraps—for example, BMRA limits the maximum copper contents of 0.20 wt% and 0.25 wt% for grades 3A and 3B, respectively—compositional variability remains a major obstacle to wider use of fragmentized scrap in EAF steelmaking. This variability mainly stems from the shredding process. For example, the shredding process of end-of-life vehicles (ELV), a major UK scrap source, mix steel from body panels with aluminum, copper wiring, and fabric interiors. Although eddy current [5] or manual sorting can reduce contaminants, tramp elements can remain attached or entangled to ferrous parts. Further shredding or manual picking can lower tramp element percentage, but applying these measures extensively is often not economically viable. Moreover, tolerance to tramp elements varies across steel grades, so many products primarily require real-time monitoring to ensure concentrations remain within acceptable limits. Consequently, continuous composition monitoring throughout recycling and steelmaking offers a cost-effective means of ensuring that steel scrap loads do not exceed tramp element thresholds, particularly for copper, which can disrupt production and comprises the desired end-product mechanical property.

While analytical tools such as X-ray fluorescence (XRF) and laser-induced breakdown spectroscopy (LIBS) can accurately measure the chemical composition of individual scrap pieces, using them to estimate the overall tramp element content of fragmentized scrap streams remains challenging. Handheld XRF and LIBS devices are constrained by per-shot acquisition times and surface sensitivity, making them ill-suited for high-throughput shredded scrap with significant piece-to-piece compositional variability. Inline XRF and LIBS scanners can detect the presence of tramp elements on moving belts, but without coupling to mass or volume measurements, they provide limited information on the overall contribution of each fragment to the bulk composition. Both technologies also face surface-related issues and require careful calibration and cleaning to obtain reliable readings on contaminated or coated scrap surfaces. In addition, the capital and operating costs of inline XRF and LIBS systems are substantial, which can hinder widespread deployment, particularly among small and medium-sized recyclers that make up a large share of the metal recycling sector.

With these challenges in mind, there is a clear need for a cost-effective method to monitor tramp element composition in order to improve the recyclability of steel scrap. With the rapid advancement of computer vision, numerous vision-based techniques have been deployed across industrial settings, supported by increasingly affordable imaging hardware. In particular, vision systems based on relatively low-cost RGB cameras offer a practical route to widespread deployment at different points in the scrap supply chain. Consequently, metal scrap recognition and quality control have emerged as active research topics, with recent work demonstrating promising results for automated scrap classification and condition assessment. In the specific context of steel scrap quality, most existing computer vision studies concentrate on geometric or morphological properties, such as scrap size or thickness, while approaches that directly infer tramp element composition from images remain scarce.

As an early contribution in this field, we would like to answer the question of whether it is feasible to use computer vision to estimate tramp element composition in commercial fragmentized steel scrap. To this end, this work focuses on copper as a representative tramp element and examines the feasibility of image-based composition estimation. Fragmentized steel scrap and copper contaminants were collected from commercially graded feedstock so that the samples reflect realistic industrial conditions. By mixing different quantities of copper with steel scrap, an image dataset was constructed that spans a range of copper weight percentages. Using image segmentation to guide the analysis, the study demonstrates that a computer vision-based approach can effectively distinguish low copper contamination from copper-rich mixtures in steel scrap.

The rest of the paper is organized as follows: the “Background” section revises the related works on computer vision-based metal recycling and scrap quality monitoring. The “Data Collection” section describes the data acquisition setup and the construction of the dataset. The experiment and its results are presented in the “Experiment” section with conclusions summarized in the “Conclusions” section.

## Background

Copper contamination is recognized as a critical constraint on the long-term circularity of steel [6]. Copper is widely distributed in end-of-life products and is difficult to remove once introduced into the melt, leading to gradual accumulation in the recycling loop. Even modest increases in copper content can lead to hot shortness and surface cracking during hot deformation, which restricts the use of scrap grades that typically contain elevated copper levels. In practice, such high-copper scrap is often only utilized after dilution with primary iron sources or large quantities of cleaner scrap to reduce the overall copper concentration to an acceptable range. Despite ongoing efforts to increase the tolerance of copper in steelmaking [7, 8], prospective assessments indicate that, under “business-as-usual” scrap management, the copper content of global scrap reserves is likely to exceed acceptable limits for many steel grades within the current century [9], underscoring the need for enhanced segregation technology as well as improve steel scrap tramp element monitoring.

Control of tramp elements such as copper currently relies predominantly on analytical techniques that provide direct measurements of chemical composition. X-ray fluorescence [10], laser-induced breakdown spectroscopy [11], and optical emission spectrometry (OES) [12] are routinely deployed in scrap yards and melt workshops to identify alloy families and quantify elemental concentrations with high accuracy. While these methods offer robust quantitative data, they are typically implemented as spot measurements, often requiring careful positioning, sufficient acquisition time, and, in some cases, surface cleaning [13], which constrains their applicability for high-throughput characterization of large volumes of heterogeneous, fragmentized scrap. As a result, only a fraction of the incoming stream may be analyzed in practice, leaving residual uncertainty in the average tramp element content of bulk scrap charges. Conveyor belt-based inline high-throughput XRF and LIBS systems are typically designed to identify the elemental signatures of individual scrap pieces or to assign them to predefined grade classes within a short detection window [14], rather than to deliver precise bulk concentration measurements, which currently makes them better suited to scrap segregation than to detailed quality monitoring. While it may be technically possible to adapt such systems for bulk concentration measurements, their capital cost and the need for controlled surface preparation on fragmentized scrap introduce additional practical challenges, which warrant further research and development before these systems can be deployed effectively for in-field use.

In recent years, rapid progress in computer vision has led to substantial advances in both network architectures and learning methodologies. Architectures have evolved from early convolutional models, such as LeNet [15], to deeper designs, such as VGG [16], skip-connected networks, such as ResNet [17] and U-Net [18], and, more recently, attention-based transformer models [19, 20], resulting in increasingly powerful neural networks that can be applied effectively to tasks including image classification, object detection, and image segmentation. As a result, computer vision-based techniques have been successfully applied to different waste material management. Such approaches have been demonstrated for plastic and drinks-container recycling, where deep learning models detect and classify items such as bottles, cans, and different polymer types on conveyor belts or in mixed-waste streams [21–23]. For aluminum recycling, machine learning and computer vision methods have been employed to distinguish between cast and wrought aluminum scrap [24, 25]. In addition, several studies have demonstrated that computer vision methods can distinguish between aluminum grades that differ in their underlying chemical composition. Huang et al. [26] used a ResNet18-based classifier to identify three grades of laboratory-prepared aluminum blocks, while Quan et al. [27] collected images of commercial pre-shredding and post-shredding aluminum scrap from the 1000, 3000, 5000, and 6000 series and showed that vision-based classifiers can exploit fine edge and texture features introduced by the shredding process to discriminate between grades. Thus, although computer vision cannot directly measure differences in chemical composition, computer vision-based methods can still infer such differences indirectly from visual cues.

There have also been rapid advances in applying computer vision to steel scrap. Han et al. [28] examined the recycling of shredded steel scrap from components such as air-conditioner and refrigerator evaporators, which contain mixed nonferrous impurities including copper and aluminum. They demonstrated that a Mask R-CNN-based model can accurately detect and classify individual scrap pieces within this mixed stream, outperforming conventional color-based methods. Tanaka et al. [29] treat nonferrous items such as motors, cables, and spray cans as anomaly classes in steel scrap, and use a fine-tuned vision–language model to detect and segment these anomalous objects. Gao et al. [30] treated the steel scrap thickness estimation as a four-class classification problem with intervals of $\le 5$ mm, $5{-}9$ mm, $9{-}12$ mm, and $\ge 12$ mm. An *F*1-score of $74\%$ is reported. Several recent studies have linked object detection with steel scrap quality assessment by exploiting geometric features such as thickness and shape. Xu et al. [31] focus on multi-category classification and rating of scrap piles using CNN-based backbones, where detected regions are used to infer grade and quality scores at the pile level. Wang et al. [32] propose a machine-vision-based steel scrap recognition model and implemented a system for scrap type and quality assessment. Their model achieves a precision of about 0.92 for steel scrap classification and 0.87 for scrap quality judgment based on overall morphology. Gao et al. [33] propose an improved YOLOv8-based object detector tailored to scrap steel images with many overlapping, irregularly shaped pieces. The proposed method managed to achieve improved performance in terms of higher average precision (AP) on a multi-shape scrap steel dataset while satisfying the computational requirement for real-time inspection.

While existing work has substantially advanced the morphological analysis of steel scrap quality, an important aspect of scrap quality, tramp element composition, remains largely unexplored in vision-based approaches. Both Han et al. [28] and Tanaka et al. [29] showed that vision models can detect copper-based scrap from ferrous scrap. These results indicate that computer vision systems are already capable of detecting tramp copper and other anomalous objects at an instance level. However, implementing weight-percentage-based quality monitoring requires linking image-based instance information with mass measurements at the batch level, and, to the best of current knowledge, no prior study has addressed this problem directly, motivating the dedicated data collection and methodology described in the following section.

## Data Collection

This work aims to evaluate whether a vision-based system can estimate the tramp copper weight percentage in mixtures of shredded steel scrap. Here, “tramp copper” denotes copper originating from discrete copper-bearing items, such as wires, motor rotors and coils (often referred to as “meatballs” by recyclers), rather than from copper intrinsically present in the shredded steel scrap itself. The underlying assumption is that, without clear visual cues, current computer vision methods are unlikely to detect small variations in bulk alloy composition from surface appearance alone, whereas they can reliably identify and quantify visually distinguishable copper-bearing objects. The work therefore restricts focus to the contribution of such visible copper objects to the total copper weight percentage and investigates whether this contribution can be inferred from image data linked to batch-level mass measurements.

**Fig. 1:**
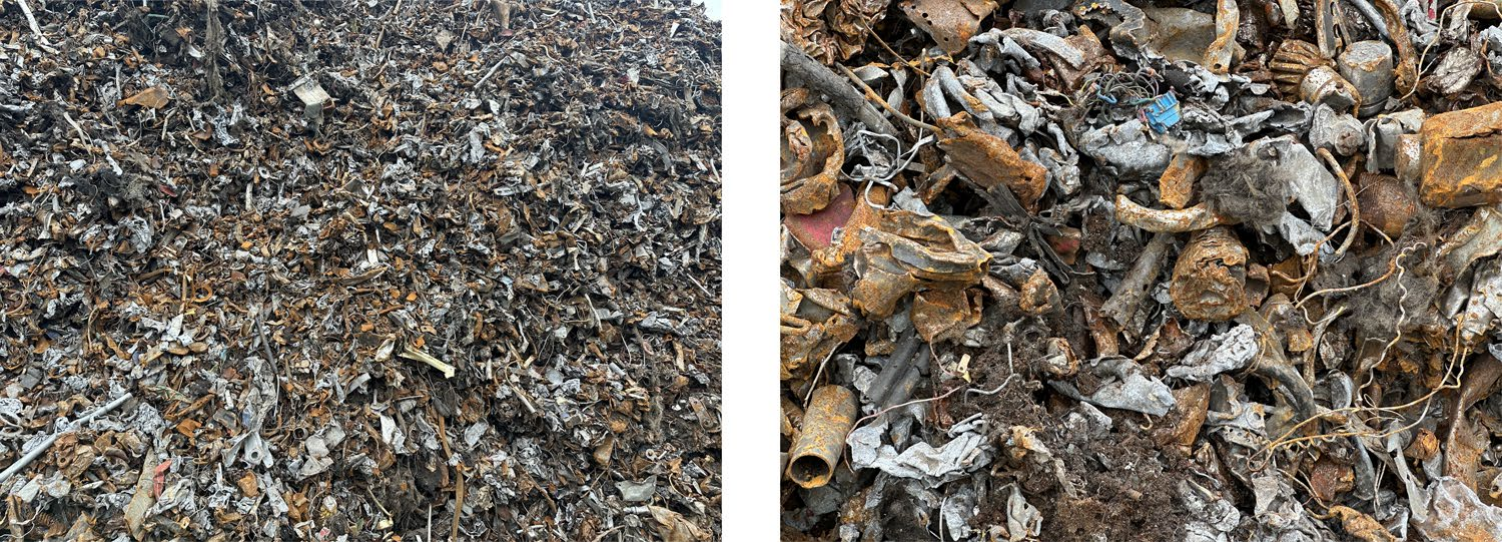
Typical appearance of British 3B grade steel scrap in a commercial scrap yard: wide field of view on the left and a magnified view on the right, where copper wires and motor rotors (“meatballs”) are clearly visible within the fragmentized scrap

**Fig. 2:**
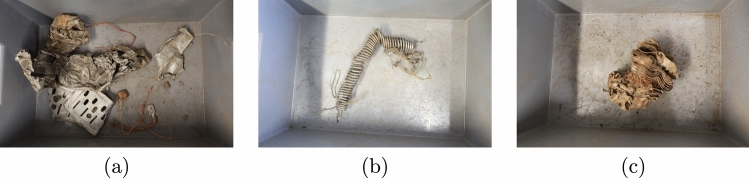
Selection of copper-bearing objects in the collected samples: **a** copper wire soldered to a ferrous component, **b** copper wire entangled with ferrous scrap during the sorting process, and **c** copper embedded within ferrous structures (“meatballs”)

The tramp copper considered in this study is restricted to wires and “meatballs”, which constitute the dominant forms of residual copper observed in British 3A and 3B grade scrap. This choice is informed by observations during site visits to commercial steel recycling plants processing end-of-life vehicles and domestic waste. Figure 1 shows photographs of 3B grade steel scrap taken in the collection pan of a commercial steel recycler. Copper residues are clearly visible in the magnified view on the right. The material has been finely shredded in accordance with the 3B specification (pieces with dimensions $\le 200$ mm in any direction), followed by eddy current separation and manual handpicking. Nevertheless, copper-bearing contaminants remain in the cleaned pile. This occurs because wires can be soldered to ferrous parts or become entangled with ferrous scrap during shredding and are difficult to detach, while rotors and coils often have heavy steel casings or silicon steel laminations that hinder their removal by eddy current separation. Examples of copper-bearing objects are shown in Fig. 2. These copper-bearing sources are particularly prevalent in waste streams such as ELVs, so, although manual picking can remove a substantial fraction of such items, a noticeable amount of copper-bearing material still remains in the sorted commercial scrap, thereby increasing the risk of tramp copper being introduced into the steelmaking process when these scraps are used.

To ensure that the collected data reflects the observed scrap quality and remains representative of commercial steel scrap in circulation, both ferrous and copper-bearing materials were sampled from commercial 3B grade scrap. To assess the feasibility of using a computer vision system to support tramp copper weight estimation, an image dataset was curated under controlled laboratory conditions. Although volumetric and weight estimation with computer vision [34, 35] remains an active research area with significant challenges, often requiring specialized sensors such as depth cameras, point-cloud acquisition, or bespoke algorithms, this study investigates whether the tramp copper weight percentage can instead be inferred from 2D segmentation maps that indicate the existence of copper-bearing objects within scrap mixtures. To link segmentation outputs with batch-level mass information, each steel scrap item and each copper-bearing piece was individually weighed. For copper wires, a subset of samples was manually unwrapped to estimate the proportion of pure copper relative to the total weight (including insulation materials such as rubber and plastic). On the basis of these measurements, an average copper content of $95\%$ by weight was adopted and applied consistently across all copper wire samples. For copper-bearing “meatballs”, full disassembly was not feasible owing to their structure. Therefore, each item was weighed as a whole, and a copper contribution of $20\%$ by weight was assumed. This estimate is consistent with industry-reported values, such as the figure by the BMRA [36]. Then, different combinations of these objects are mixed to produce scrap batches spanning a range of tramp copper weight percentages.

**Fig. 3:**
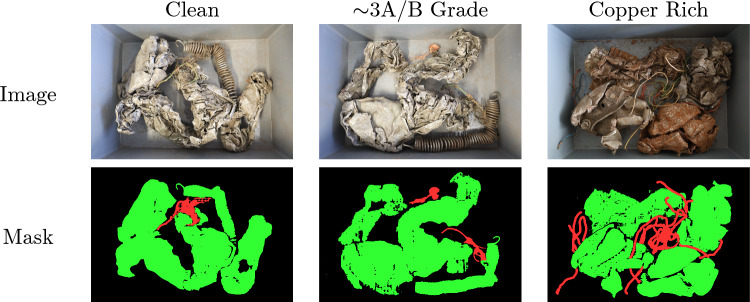
Example scrap mixtures for each copper composition class with corresponding ground-truth semantic segmentation masks: columns show samples from the clean, $\sim$ 3A/B grade, and copper-rich classes respectively. Rows show RGB images (top) and pixel-wise masks (bottom) with three labels indicating ferrous scrap, copper-bearing objects, and empty background

We treat the copper weight-percentage estimation problem as a three-class classification task, with class 1 being “clean” for copper $\le 0.1$ wt%, class 2 between 0.1 and 0.25 wt% for “$\sim$ 3A/B grade”, and class 3 “copper rich” for copper $\ge 0.25$ wt%. The choice of these three copper composition classes follows the BMRA copper weight-percentage limits for grades 3A and 3B fragmentized scrap. For each batch, ferrous and copper-bearing scrap pieces are mixed in a box of fixed dimensions, which serves as a reference frame for both image acquisition and apparent scrap size. A Canon R10 digital camera with a 16-mm lens is mounted on a tripod directly above the box for image acquisition, ensuring consistent imagery throughout the dataset collection process. Images are taken in JPEG format with the resolution of $6000\times 4000$ pixels. A total of 200 images per class are collected, with batch weights recorded and per-pixel segmentation masks manually annotated. In addition, 416 further images with segmentation masks only (no associated weight measurements) are acquired to provide extra supervision for the segmentation network and improve its accuracy. The semantic segmentation task is defined over three classes: ferrous scrap, copper-bearing objects, and background. During annotation, a pre-trained segment anything model (SAM) [37] is first employed to generate candidate object contours, after which a single annotator refines these contours and assigns a final class label to each region. Sample images together with their corresponding segmentation masks are provided in Fig. 3.

## Experiment

Using the collected images, their semantic segmentation, and the corresponding batch weight measurements, we conducted experiments to assess whether this setup can be used to infer the copper weight-percentage range in steel scrap as a classification task. To evaluate the benefit of segmentation, we compare classification performance under three configurations: decisions based on RGB images only, RGB images combined with inferred segmentation maps, and RGB images combined with oracle segmentation maps. The collected 600 images with associated weight measurements are split into 510 training images and 90 test images. It is important to note that a single scrap object may appear in multiple images. As the dataset is randomly split into training and test sets, the same physical object may therefore be present in both. To ensure a natural distribution of the scraps, the objects are placed in the box and then mixed through mechanical agitation prior to image acquisition. Owing to the highly irregular shapes of the shredded scraps and “meatballs”, and the flexibility of the copper wires, this process results in substantial variation in their positions, orientations, and spatial arrangements relative to the fixed top-view camera. Consequently, even when the same physical object appears across different images, its visual appearance can vary substantially. By introducing significant variability of the objects’ appearance across different images, this reduces the risk of the model overfitting to object identity and encourages the model to learn more generalizable visual features, while leveraging meticulously measured weight information associated with each object.

### Image Segmentation

To infer the semantic segmentation maps, a vision transformer (ViT)-based segmentation model is employed. The network is trained using the 510 images with associated weight measurements together with 380 of the additional 416 images that have segmentation labels only, resulting in a total of 890 training images with pixel-wise annotations. Given that the original images have a resolution of $6000 \times 4000$ pixels, processing the images is computationally demanding, particularly in the context of real-time scrap monitoring. We therefore uniformly downscale them to $600\times 400$ pixels, which preserves sufficient detail for object delineation while keeping memory and runtime requirements manageable on moderately specified hardware. The ViT model is trained on a workstation equipped with an NVIDIA RTX 3090 GPU with 24 GB of VRAM, using standard data augmentation techniques including random rotations, horizontal flips, and additive Gaussian noise to improve robustness and reduce overfitting. The code is implemented using PyTorch. Evaluated on a reserved test set of 36 images, the model achieves a mean intersection of union (mIoU) of $72\%$. The per-class precision, recall and IoU are provided in Table 1.

**Table 1:** Segmentation performance for ferrous and copper-bearing classes

Class	IoU	Precision	Recall
Ferrous scrap	0.77	0.86	0.88
Copper-bearing	0.59	0.64	0.88

**Fig. 4:**
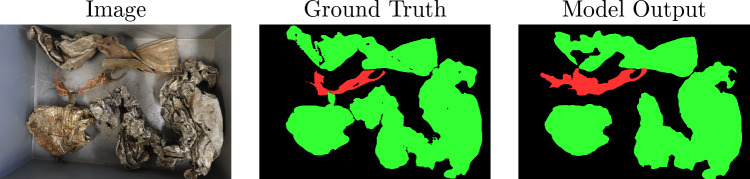
An example of the comparison between the ground truth and model output from the trained segmentation model

While the quantitative performance leaves room for further improvement, the predicted masks provide a practically useful decomposition of the scene into ferrous and copper-bearing regions. Visual inspection confirms that the model reliably captures the majority of copper-bearing components across a wide range of scrap sizes, surface textures, and illumination conditions, as illustrated in Fig. 4. Although there are local discrepancies between the predicted and ground truth masks, the network consistently recovers all major ferrous and copper-bearing objects, indicating that the representation learned by the vision transformer is well aligned with the downstream objective of tramp copper estimation. A further contributor to the nonperfect mIoU is the presence of annotation noise: irregular scrap geometries, overlapping pieces, shadows, and low-contrast regions all introduce ambiguity in boundary placement, and small misalignment from the SAM-based annotation pipeline can systematically reduce overlap scores even when object-level detection is correct. This is further supported by the numeric results in Table 1, where the copper-bearing class exhibits lower IoU and precision than the ferrous scrap class, reflecting the more irregular geometries of copper-bearing objects such as wires, which also complicate annotation. Nevertheless, the high recall indicates that the model still reliably detects the copper-bearing objects in the images. In this context, exact pixel-wise agreement with the ground truth masks, which would yield very high mIoU values, is neither realistic nor necessary; instead, segmentation quality is better judged by its impact on the subsequent tramp copper weight estimation, evaluated in the next subsection.

### Tramp Copper Weight Percentage Estimation

The weight-percentage prediction problem is formulated as a three-class image classification task, with 510 images used for training and 90 images held out for testing. To analyze the effect of segmentation cues, three separate ViT classifiers are trained under different input configurations: (i) RGB images combined with oracle (ground truth) masks, (ii) RGB images combined with inferred masks from the segmentation network, and (iii) RGB images only, referred to as the *oracle*, *segmentation*, and *RGB-only* models, respectively. The RGB-only model is included as a minimal baseline to isolate the contribution of segmentation cues.

#### Network Architecture and Training Setup

The network backbone of the classification task is a vision transformer (ViT) with an input size of $600 \times 400$ with a patch size of $24 \times 16$. The ViT backbone used an embedding dimension of 512, 8 transformer layers, 16 attention heads, and an MLP dimension of 2048. The classification head was replaced by a fully connected layer with three output neurons, followed by a softmax activation, to predict the three weight-percentage classes. For the RGB-only setting, the model received a three-channel input image. For the oracle- and segmentation-based settings, the RGB image was concatenated with the three-channel class mask, producing a six-channel input tensor. In these cases, the first ViT projection layer was adapted accordingly to accept six input channels, while all other architectural settings remained unchanged.

**Table 2:** Summary of images and input dimensions used across experiments

Stage	Train/test	Input (Channels)	Size (pixels)
Segmentation	890/36	RGB (3)	$600\times 400$
Cu% classif. (RGB-only)	510/90	RGB (3)	$600\times 400$
Cu% classif. (inferred mask)	510/90	RGB + mask (6)	$600\times 400$
Cu% classif. (oracle mask)	510/90	RGB + mask (6)	$600\times 400$

**Fig. 5:**
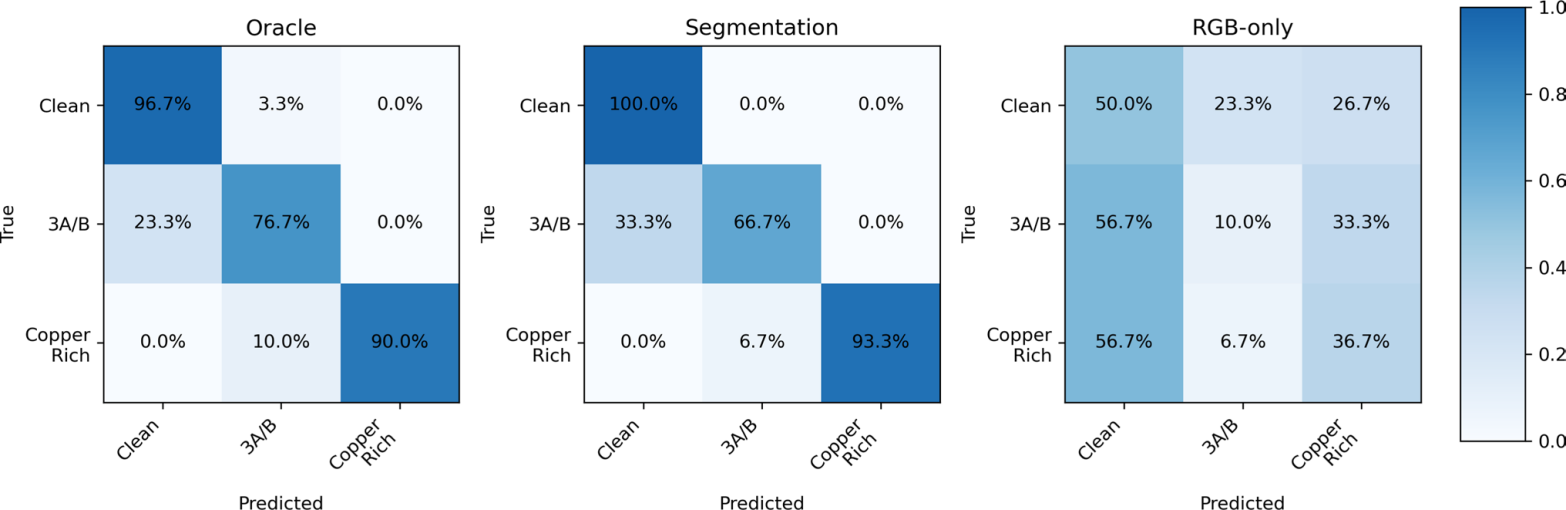
Confusion matrices summarizing the test-set performance of the three classifiers. From left to right: the panels correspond to the “oracle”, “segmentation”, and “RGB-only” models

During training, standard data augmentation is applied to the RGB channels for all three tasks, including random rotations, horizontal flips, and additive Gaussian noise. For the oracle model, the same geometric transformations are synchronously applied to the ground truth masks to preserve alignment with the augmented RGB images. For the segmentation model, the RGB image is first augmented, then passed through the trained segmentation network to obtain an inferred mask, and finally the augmented RGB and corresponding mask are stacked to produce the classifier input, enabling an end-to-end pipeline that only requires an RGB image at inference time. In all three configurations, the ViT partitions the $600\times 400$ input into nonoverlapping $24\times 16$ pixel patches, which are treated as tokens by the transformer encoder, enabling the attention mechanism to operate at the patch level across the entire image. For the training process, we optimized the network using RMSprop with a learning rate of $1 \times 10^{-5}$, a batch size of eight, and 1000 training epochs. The loss function was cross-entropy loss. A summary of the image datasets and their input configurations is given in Table 2.

#### Experimental Results

1 $$ {\text {Precision}} = \frac{{\text {TP}}}{{\text {TP}} + {\text {FP}}}, $$

2 $$ {\text {Recall}} = \frac{{\text {TP}}}{{\text {TP}} + {\text {FN}}}, $$

3 $$ {F1} = \frac{2\,{\text {Precision}}\,{\text {Recall}}}{{\text {Precision}} + {\text {Recall}}}, $$

4 $$ {\text {FPR}} = \frac{{\text {FP}}}{{\text {FP}} + {{\text {TN}}}}. $$

**Table 3:** Per-class precision, recall, *F*1-score and false-positive rate (FPR) for the three classifiers on the test set

Model	Class	Precision	Recall	*F*1	FPR
Oracle	Clean	0.81	0.97	0.88	0.12
	3A/B	0.85	0.77	0.81	0.07
	Copper rich	1.00	0.90	0.95	0.00
Segmentation	Clean	0.75	1.00	0.86	0.17
	3A/B	0.91	0.67	0.77	0.03
	Copper rich	1.00	0.93	0.97	0.00
RGB-only	Clean	0.31	0.50	0.38	0.57
	3A/B	0.25	0.10	0.14	0.15
	Copper rich	0.38	0.37	0.37	0.30

Evaluated on the test set, the confusion matrices for the three ViT-based classifiers are shown in Fig. 5. We also measure the precision, recall, *F*1-score, and false-positive rates for the tested results in Table 3, with metric definitions given in Eqs. 1–4, where ${\text {TP, FP, FN}}$ and ${\text {TN}}$ denote the number of true positives, false positives, false negatives, and true negatives, respectively, and all metrics are computed per class. The RGB-only model exhibits weak discriminative power for copper weight percentage: a large fraction of samples are pushed toward the extreme classes (“clean” or “copper rich”), resulting in an overall accuracy of only $32.22\%$ and low per-class *F*1-scores. This behavior is not unexpected with the difficulty of inferring bulk copper content directly from RGB appearance alone, particularly given the limited dataset size and the fact that the visual signal associated with copper weight is indirect and often subtle. The RGB-only classifier fails to consistently associate the presence and extent of copper-bearing objects in the scene with the underlying weight-percentage labels.

By contrast, the oracle and segmentation-guided classifiers achieve substantially higher and closely matched accuracies of $87.78\%$ and $86.67\%$, respectively. The per-class *F*1 scores are consistently higher than the results from RGB-only. Incorporating segmentation masks, either as ground-truth annotations or as predictions from the segmentation network, allows the classifier to exploit explicit information about the location and extent of copper-bearing regions, leading to substantially improved performance compared with using RGB cues alone. The comparable performance of the oracle and segmentation models indicates that, even though the inferred masks do not perfectly coincide with the ground truth, they capture the dominant ferrous and copper-bearing structures sufficiently well for reliable class discrimination.

The zero FPRs of the oracle and segmentation-based models are encouraging, even though they are obtained from a relatively small dataset. These results indicate that the models can consistently and reliably identify copper-rich classes against the other categories, enabling a low rejection rate for batches that fall within the specified standard. The remaining errors for both segmentation-based classifiers are dominated by confusion between the “$\sim$ 3A/B” and “clean” classes, where images with very low but nonzero copper content are occasionally assigned to the clean category. This indicates that the most challenging setting is discriminating very small differences in copper weight percentage, which likely calls for more finely resolved annotations, additional training data in the boundary regime, or auxiliary cues that better emphasize small copper-bearing fragments. Nonetheless, the findings show that segmentation-guided classification can potentially provide a practical approach to estimating copper weight-percentage from relatively modest datasets.

## Conclusions

This study demonstrates that computer vision, and in particular segmentation-guided image analysis, can be a feasible approach to estimating tramp copper weight percentage in fragmentized steel scrap. The problem is particularly acute in the UK, where the shift toward scrap-based electric arc furnace steelmaking increases reliance on 3A/3B-grade fragmentized scrap and amplifies sensitivity to tramp element variability in incoming material streams. Conventional analytical techniques, including handheld or inline XRF and LIBS, remain well suited for spot analysis of individual pieces or for segregation rather than continuous quality monitoring. Handheld XRF and LIBS are limited by low throughput, while inline systems face high costs that prevent their widespread adoption. Both approaches require more in-depth integration with bulk mass information to function as continuous monitors of batch-level copper content in highly heterogeneous scrap mixtures. Against this backdrop, the findings of this work indicate that vision-based models leveraging semantic segmentation can help bridge this gap by relating the presence of visually identifiable copper-bearing objects to copper weight-percentage ranges at the batch level.

By combining RGB images with either oracle or inferred segmentation masks, the proposed classifiers successfully distinguish between “clean”, “$\sim$ 3A/B-grade”, and “copper-rich” mixtures with substantially higher accuracy than an RGB-only baseline. The segmentation information focuses the classifier on the spatial distribution and extent of copper-bearing objects, allowing it to associate these features with batch-level weight-percentage labels in a way that purely appearance-based models struggle to achieve, especially when trained on modest datasets. The comparable performance of the oracle and segmentation-guided models further indicates that, for this task, approximate but consistent segmentation of ferrous and copper-bearing regions is sufficient to yield robust classification, even in the presence of annotation noise and imperfect mask boundaries. These findings highlight segmentation-guided classification as a potential component within a broader toolkit for tramp element monitoring in industrial steel recycling.

As a feasibility study, the present experiments are carried out under controlled laboratory conditions, using material sampled from commercial 3B scrap but imaged in a fixed setup with a fixed viewpoint and placed in a box of fixed size, which also serves as a size reference. Within this setting, the segmentation model attains a good mean intersection over union on reserved data but still exhibits some local inaccuracies in complex regions. For the weight percentage classification, the most challenging cases occur near the boundary between the “clean” and “$\sim$ 3A/B-grade” classes, where very small copper-bearing fragments or thin wires are visually subtle yet can alter the classification outcome. These observations underscore the importance of further improving mask quality, particularly through more detailed pixel-level annotation around fine copper structures and ambiguous boundaries, in order to sharpen discrimination in low-copper regimes.

Building on the encouraging laboratory results, future work may investigate the robustness of the proposed framework under more challenging imaging conditions, such as variable lighting, motion blur, occlusions, and broader scrap geometries. Such extensions would likely require additional domain adaptation and data augmentation. Future studies may also explore incorporating batch-level mass measurements and process data to support more comprehensive copper-content estimation, as well as extending the framework to multi-element analysis by considering other tramp elements and more fine-grained scrap categories.

Finally, the proposed framework may be complementary to existing analytical methods and could potentially be integrated with them in future studies. For example, segmentation-based detection of copper-rich or otherwise uncertain regions may help identify cases where follow-up XRF or LIBS measurements would be most informative. In such a setting, vision would act as a preliminary screening tool, while analytical measurements could provide additional confirmation for selected samples or ambiguous cases. Overall, the results presented in this work provide an encouraging basis for further investigation of segmentation-guided monitoring of tramp copper in fragmentized steel scrap.

## References

[1] Hasanbeigi A (2022) Steel climate impact. An international benchmarking of energy and CO intensities. Global Efficiency Intelligence, St. Petersburg

[2] Zang G, Sun P, Elgowainy A, Bobba P, McMillan C, Ma O, Podkaminer K, Rustagi N, Melaina M, Koleva M (2023) Cost and life cycle analysis for deep CO emissions reduction of steelmaking: blast furnace–basic oxygen furnace and electric arc furnace technologies. Int J Greenh Gas Control 128:103958. 10.1016/j.ijggc.2023.103958

[3] British Metals Recycling Association (2023) UK specifications for metals recycling for ferrous raw materials, 3rd edn. British Metals Recycling Association, Huntingdon (2023). https://www.recyclemetals.org/newsandarticles/updated-ferrous-specification-booklet.html

[4] Hall R, Zhang W, Li Z (2021) Domestic scrap steel recycling—economic, environmental and social opportunities (EV0490). DEFRA, London

[5] Kercher S, Webb M (1982) Scrap processing by eddy current separation techniques. Resour Conserv 8(1):61–74

[6] Daehn KE, Cabrera Serrenho A, Allwood JM (2017) How will copper contamination constrain future global steel recycling? Environ Sci Technol 51(11):6599–6606 28445647 10.1021/acs.est.7b00997

[7] Duan J, Farrugia D, Davis C, Yan Z, Li Z (2023) Synergistic effect of residual elements on oxidation rates and oxide/metal interface characteristics in a low-carbon steel oxidized at C for 3 hours. Ironmak Steelmak 50(11):1559–1570

[8] Kapoor I, Davis C, Li Z (2024) Effect of residual elements during the hot-working process of steel production: a critical review. Steel Res Int 95(9):2400116

[9] Loibl A, Tercero Espinoza LA (2021) Current challenges in copper recycling: aligning insights from material flow analysis with technological research developments and industry issues in Europe and North America. Resour Conserv Recycl 169:105462. 10.1016/j.resconrec.2021.105462

[10] Al-Eshaikh MA, Kadachi A (2011) Elemental analysis of steel products using X-ray fluorescence (XRF) technique. J King Saud Univ Eng Sci 23(2):75–79

[11] Vieitez MO, Hedberg J, Launila O, Berg L-E (2005) Elemental analysis of steel scrap metals and minerals by laser-induced breakdown spectroscopy. Spectrochim Acta B 60(7–8):920–925

[12] Pande MM, Guo M, Dumarey R, Devisscher S, Blanpain B (2011) Determination of steel cleanliness in ultra low carbon steel by pulse discrimination analysis-optical emission spectroscopy technique. ISIJ Int 51(11):1778–1787

[13] Bröms D, Khan MI (2024) Laser-induced breakdown spectroscopy for enhanced scrap recycling. KTH Royal Institute of Technology, Stockholm

[14] Pedarnig JD, Trautner S, Grünberger S, Giannakaris N, Eschlböck-Fuchs S, Hofstadler J (2021) Review of element analysis of industrial materials by in-line laser-induced breakdown spectroscopy (LIBS). Appl Sci 11(19):9274

[15] LeCun Y, Bottou L, Bengio Y, Haffner P (2002) Gradient-based learning applied to document recognition. Proc IEEE 86(11):2278–2324

[16] Simonyan K, Zisserman A (2014) Very deep convolutional networks for large-scale image recognition. arXiv preprint. arXiv:1409.1556

[17] He K, Zhang X, Ren S, Sun J (2016) Deep residual learning for image recognition. In: Proceedings of the IEEE conference on computer vision and pattern recognition, 2016, pp 770–778

[18] Ronneberger O, Fischer P, Brox T (2015) U-Net: convolutional networks for biomedical image segmentation. In: International conference on medical image computing and computer-assisted intervention, 2015. Springer, pp 234–241

[19] Vaswani A, Shazeer N, Parmar N, Uszkoreit J, Jones L, Gomez AN, Kaiser Ł, Polosukhin I (2017) Attention is all you need. In: Advances in neural information processing systems, 2017, vol 30

[20] Dosovitskiy A (2020) An image is worth words: transformers for image recognition at scale. arXiv preprint. arXiv:2010.11929

[21] Serezhkin A (2020) Drinking waste classification. Kaggle

[22] Martinez-Hernandez U, West G, Assaf T (2024) Low-cost recognition of plastic waste using deep learning and a multi-spectral near-infrared sensor. Sensors 24(9):2821 38732925 10.3390/s24092821 PMC11086069

[23] Xie S, Wu H, Mao W, Chu X, Meng Y, Yang X (2025) Study on efficient recognition and accurate localization method of waste plastic bottles based on deep learning. Ecol Inform 86:103020

[24] Díaz-Romero D, Sterkens W, Van den Eynde S, Goedemé T, Dewulf W, Peeters J (2021) Deep learning computer vision for the separation of cast- and wrought-aluminum scrap. Resour Conserv Recycl 172:105685

[25] Williams KC, O’Toole MD, Peyton AJ (2024) Classification of wrought and cast aluminium using magnetic induction spectroscopy and machine vision. In: 2024 IEEE sensors applications symposium (SAS), 2024, pp 1–6. 10.1109/SAS60918.2024.10636579

[26] Huang B, Liu J, Zhang Q, Liu K, Li K, Liao X (2022) Identification and classification of aluminum scrap grades based on the ResNet18 model. Appl Sci 12(21):11133

[27] Quan Y, Dunn M, Montana G, Li Z (2025) Computer vision-based aluminum scrap grade classification and detection for upcycling. J Sustain Metall 11(3):2954–2964

[28] Han SD, Huang B, Ding S, Song C, Feng SW, Xu M, Lin H, Zou Q, Boularias A, Yu J (2021) Toward fully automated metal recycling using computer vision and non-prehensile manipulation. In: 2021 IEEE 17th international conference on automation science and engineering (CASE), 2021. IEEE, pp 891–898

[29] Tanaka D, Karasawa T, Takenouchi S, Kawakami R (2025) Anomaly object segmentation with vision-language models for steel scrap recycling. arXiv preprint. arXiv:2506.13282

[30] Gao Z, Lu H, Lei J, Zhao J, Guo H, Shi C, Zhang Y (2023) An RGB-D-based thickness feature descriptor and its application on scrap steel grading. IEEE Trans Instrum Meas 72:1–1437323850

[31] Xu W, Xiao P, Zhu L, Zhang Y, Chang J, Zhu R, Xu Y (2023) Classification and rating of steel scrap using deep learning. Eng Appl Artif Intell 123:106241

[32] Wang L, Xu Y, Li R, Yang S, Liu P, Li H (2025) A steel scrap recognition model based on machine vision. J Meas Eng. 10.21595/jme.2024.24321

[33] Gao Y, Liu W, Chui H-C, Chen X (2024) Large span sizes and irregular shapes target detection methods using variable convolution-improved YOLOv8. Sensors 24(8):2560 38676177 10.3390/s24082560 PMC11054827

[34] Kamari M, Ham Y (2021) Vision-based volumetric measurements via deep learning-based point cloud segmentation for material management in jobsites. Autom Constr 121:103430

[35] Standley T, Sener O, Chen D, Savarese S (2017) image2mass: estimating the mass of an object from its image. In: Conference on robot learning, 2017. PMLR, pp 324–333

[36] BMR Association (2026) BHS-Sonthofen increases copper recovery. BMR Association. https://www.recyclemetals.org/newsandarticles/bhs-sonthofen-rotorshredder-copper-recovery.html. Accessed 22 May 2026

[37] Kirillov A, Mintun E, Ravi N, Mao H, Rolland C, Gustafson L, Xiao T, Whitehead S, Berg AC, Lo W-Y (2023) Proceedings of the IEEE/CVF international conference on computer vision, 2023, pp 4015–4026

